# Innate Immune Basis for Rift Valley Fever Susceptibility in Mouse Models

**DOI:** 10.1038/s41598-017-07543-8

**Published:** 2017-08-02

**Authors:** Rashida Lathan, Dominique Simon-Chazottes, Grégory Jouvion, Ophélie Godon, Marie Malissen, Marie Flamand, Pierre Bruhns, Jean-Jacques Panthier

**Affiliations:** 1 Department of Developmental & Stem Cell Biology, Unit of Mouse Functional Genetics, Institut Pasteur, F-75015 Paris, France; 2 Centre National de la Recherche Scientifique, CNRS UMR 3738, F-75015 Paris, France; 3 Department of Infection & Epidemiology, Unit of Human Histopathology and Animal Models, Institut Pasteur, F-75015 Paris, France; 4 Department of Immunology, Unit of Antibodies in Therapy & Pathology, Institut Pasteur, F-75015 Paris, France; 5 Institut National de la Santé et de la Recherche Médicale, INSERM U1222, F-75015 Paris, France; 6 Centre d’Immunologie de Marseille-Luminy, Aix Marseille Université UM2, F-13288 Marseille, France; 7 Institut National de la Santé et de la Recherche Médicale, INSERM U1104, F-13288 Marseille, France; 8 Centre National de la Recherche Scientifique, CNRS UMR7280, F-13288 Marseille, France; 9 Department of Virology, Unit of Structural Virology, Institut Pasteur, F-75015 Paris, France

## Abstract

Rift Valley fever virus (RVFV) leads to varied clinical manifestations in animals and in humans that range from moderate fever to fatal illness, suggesting that host immune responses are important determinants of the disease severity. We investigated the immune basis for the extreme susceptibility of MBT/Pas mice that die with mild to acute hepatitis by day 3 post-infection compared to more resistant BALB/cByJ mice that survive up to a week longer. Lower levels of neutrophils observed in the bone marrow and blood of infected MBT/Pas mice are unlikely to be causative of increased RVFV susceptibility as constitutive neutropenia in specific mutant mice did not change survival outcome. However, whereas MBT/Pas mice mounted an earlier inflammatory response accompanied by higher amounts of interferon (IFN)-α in the serum compared to BALB/cByJ mice, they failed to prevent high viral antigen load. Several immunological alterations were uncovered in infected MBT/Pas mice compared to BALB/cByJ mice, including low levels of leukocytes that expressed type I IFN receptor subunit 1 (IFNAR1) in the blood, spleen and liver, delayed leukocyte activation and decreased percentage of IFN-γ-producing leukocytes in the blood. These observations are consistent with the complex mode of inheritance of RVFV susceptibility in genetic studies.

## Introduction

Rift Valley Fever (RVF) is caused by an emerging arbovirus endemic in sub-Saharan African countries. RVF virus (RVFV) may spread as a result of the movement of infected animals. Epizootics are identified by a large number of mass abortions, perinatal mortality and hemorrhagic syndrome in livestock^1^. In a minority of infected humans, the disease progresses from a self-limiting febrile illness to severe hepatitis with hemorrhagic manifestations, encephalitis, and ocular lesions^2, 3^. The RVFV infection in laboratory rodents mimics many aspects of the pathology in humans^4, 5^. In particular, infection of BALB/c mice recapitulates the hepatitis and encephalitis observed in human disease^6^. The mouse liver is an early and dominant target of RVFV, with extensive damage to hepatocytes via apoptosis. Mice that survived this early hepatic phase develop infection in the brain. Lymphoid tissues are also affected by RVFV. The main lymphoid lesion is lymphocyte apoptosis (lymphocytolysis) as observed in the thymus, spleen, lymph nodes, and mucosa-associated lymphoid tissues^6, 7^. Moreover, a spectrum of pathogenic phenotypes can be observed in inbred mice, as in humans^6, 8–10^, suggesting that host genetic factors are important determinants of the susceptibility to RVF disease. We have previously shown that wild-derived inbred MBT/Pas (MBT) mice are highly susceptible to infection with the virulent RVFV ZH548 and Kenya 98 strains in comparison to more resistant BALB/cByJ (BALB/c) mice^11^. We have demonstrated that the susceptibility in MBT mice is a complex trait, inherited in a multifactorial manner^12^. Three different quantitative trait loci (QTLs) have been associated with the severity of the disease. The effects of these QTLs are accumulative yet modest, as they explain only 8.3% of the difference in susceptibility between BALB/c and MBT mice. This implies that susceptibility is a result of several independent mechanisms controlled by many DNA variants with minor effects^12^. Mouse embryonic fibroblasts (MEFs) derived from MBT mice elicited a delayed and partial type I IFN response when infected with RVFV^11^. This result suggested that MBT mice fail to induce, in due course, a complete innate immune response which contributes to their susceptibility to RVF^11^.

Innate immune cells, and in particular cell subsets such as dendritic cells (DCs), natural killer (NK) cells, and neutrophils play crucial roles in early antiviral defense. Though DCs are not involved in early pathogen clearance, they are potent antigen-presenting cells and are a source of viral protective type I interferons (IFNs)^13^. Type I IFNs response is precipitated through the IFN-α and -β (IFNAR1/2) heterodimeric receptor, and the downstream induction of IFN-stimulated genes (ISGs) is responsible for an effective antiviral defense. It has been suggested that the cell surface expression level of IFNAR1 is a determining factor for specific cellular responses^14^. NK cells are early viral sensors that lyse virus-infected cells, and regulate the adaptive immune response by secreting cytokines such as IFN-γ^15^. Defects in NK cell activity, such as decreased production of IFN-γ, could render mice more susceptible to viral infection^16^. Neutrophils are abundant, highly motile, and efficient phagocytic immune cells. It has been suggested that the key to their active role during infection in mice is an ability to be replenished through rapid proliferation within the bone marrow^17^. Though neutrophils are more commonly associated with defense against bacterial and fungal pathogens, they have been demonstrated to play protective roles in mice against influenza virus during the initial stages of the innate immune response in limiting virus replication and spread, and lethality^18^. Despite their individual contributory roles in viral protection, innate immune cells act rarely in isolation, as crosstalk and interaction characterize innate immune protection in mammalian hosts. DCs, NK cells, and neutrophils have all been reported to interact during viral resistance and collectively create a microenvironment of IFNs, cytokines, and cell activation required in viral clearance^19^.

RVFV is sensitive to the action of type I IFNs, but it has evolved a comprehensive mechanism to limit their effects by maintaining the promoter of IFN-β (*Ifnb1*) gene in a repressed state^20, 21^ (see recent review in ref. 22). Several studies provided evidence that RVFV evades the innate immune response, thus facilitating infection of hepatic cells at days 3–5 post infection^9, 10^. In this context, the role of innate immune cells in RVFV infection is not understood.

The study described here was developed with the intent to establish the contributions of innate immune cells in the susceptible MBT and more resistant BALB/c mice after infection with virulent wild type RVFV. We investigated the innate immune cell population fluctuations within the organ compartments, including those of high viral antigen load, the blood, spleen and liver, and evaluated the expression of viral sensing components (IFN-α and -β receptor 1 [IFNAR1]), and migratory and activation markers (P-selectin glycoprotein ligand-1 [PSGL-1], and IFN-γ). Herein, we present evidence that RVFV-infected MBT mice display a number of immune-related defects in the multi-faceted antiviral response.

## Results

### Host genetics determines degree of susceptibility to RVFV-induced hepatitis

**Figure 1:**
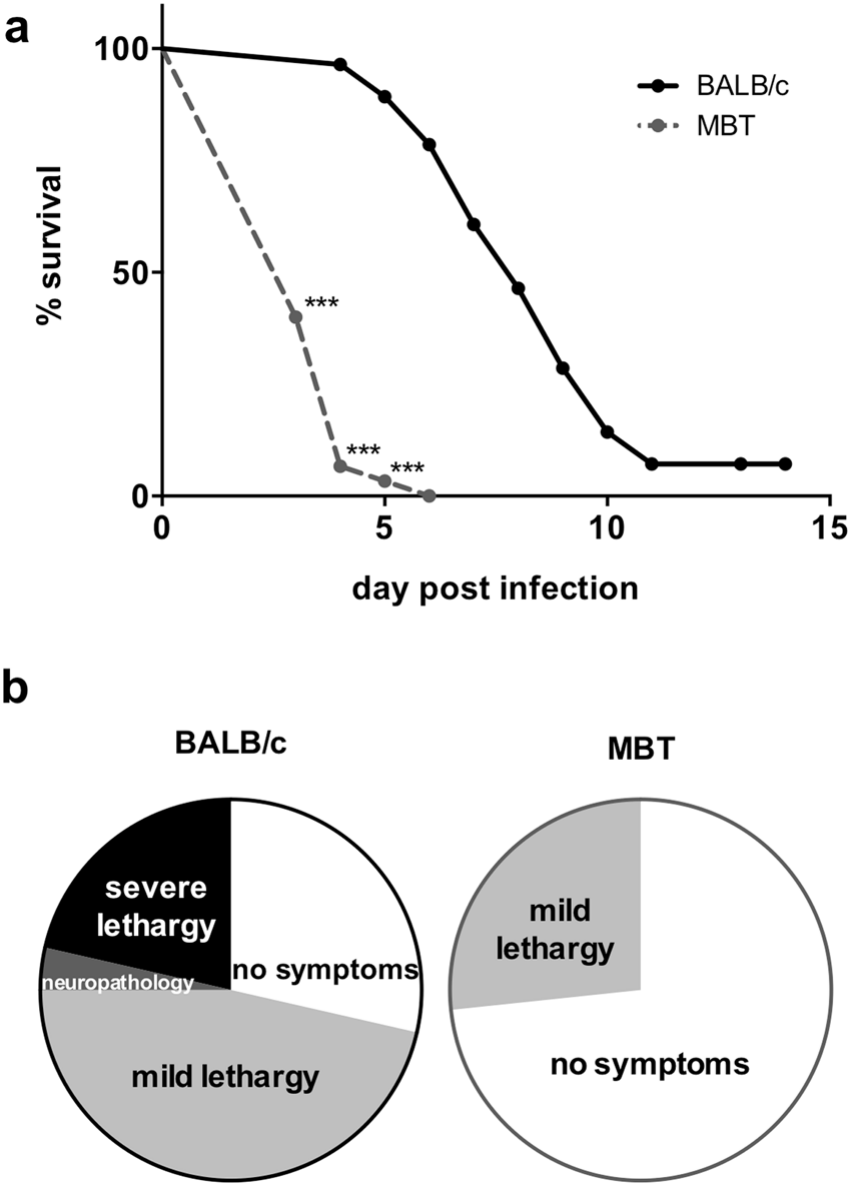
Host genetics contributes to susceptibility and symptoms of RVF disease. **(a)** Survival curves of BALB/c (n = 28) and MBT (n = 30) mice infected intraperitoneally with 10^2^ PFU RVFV strain ZH548. **(b)** Trajectory of symptoms of these mice as observed daily, preceding death. Mantel-Cox’s Logrank test was performed to assess survival curve differences. ***P < 0.001.

Groups of 10 to 20 mice from six inbred strains (C57BL/6, BALB/c, PWK/Pas, SEG/Pas, 129/SvPas, and MBT) were infected by RVFV to create mouse models to define the inherited determinants of antiviral host defense in susceptible and resistant mice. Mice were monitored for survival time and clinical outcome. Results uncovered extreme survival phenotypes between a highly susceptible wild-derived strain, MBT, and a more resistant classical laboratory strain, BALB/c^11, 23^. BALB/c mice died at a median of 8 days, and 3 out of 28 (11%) survived past 12 days post infection with 100 plaque-forming units (PFU) of RVFV strain ZH548. By contrast, 30 out of 30 (100%) MBT mice died at a median of 3 days, and none survived past 6 days post infection (Fig. 1a). In addition, BALB/c mice underwent a complete spectrum of symptoms that included a slow gait, clinical signs of neuropathology with lower limb paralysis and/or head tilting and spinning, and severe lethargy. In contrast, the clinical symptoms of MBT mice before death were acute with the majority of animals displaying no physical representation of disease, and only 27% displaying a slow gait within 24 h preceding death (Fig. 1b).

**Figure 2:**
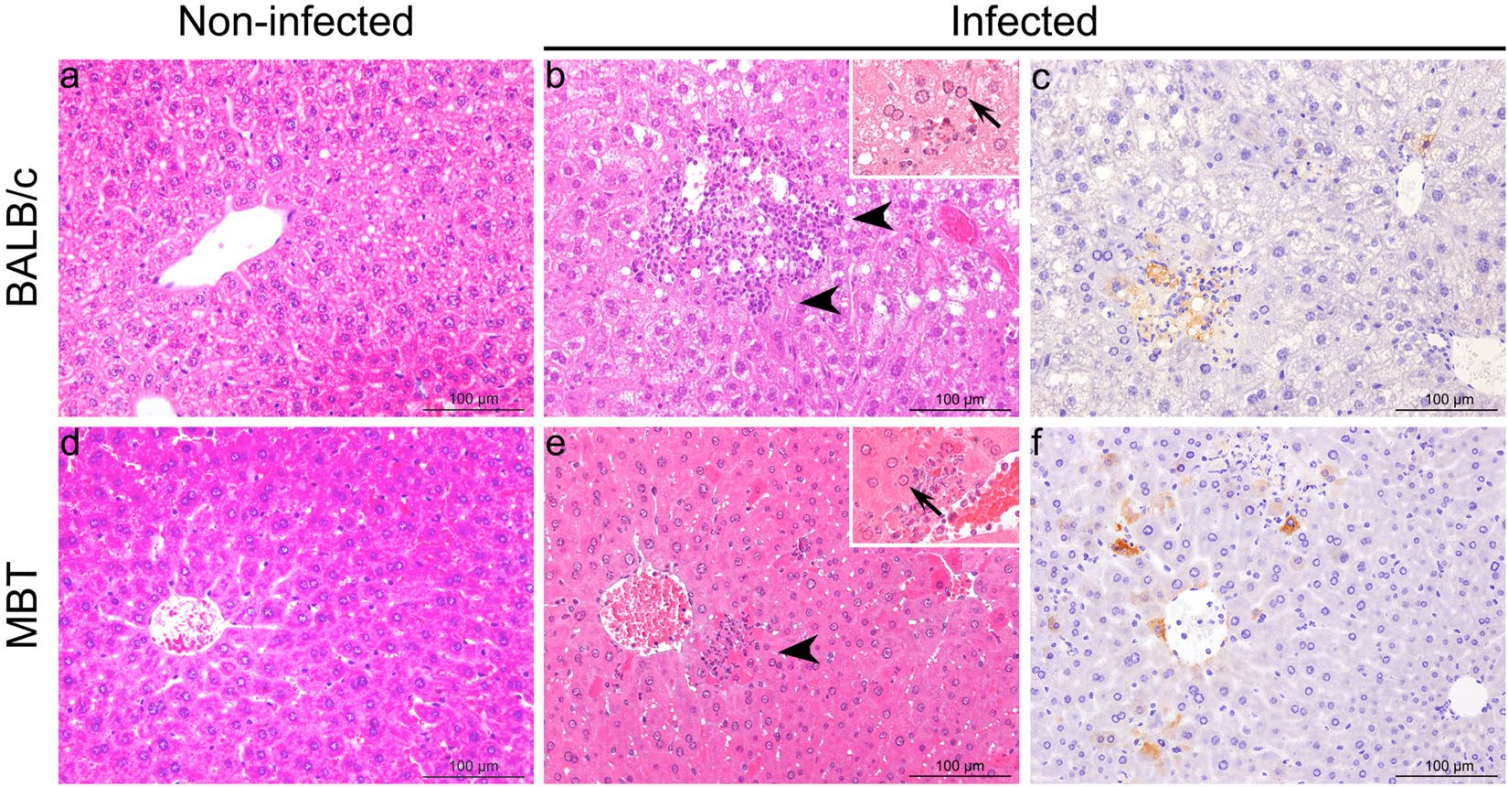
Histopathological analysis from non-infected and RVFV-infected BALB/c and MBT mice at day 3 post infection. (**a**,**b**,**d**,**e**) Hematoxylin and eosin (HE) staining of liver: arrowheads indicate lesions with necrotic and apoptotic hepatocytes, and neutrophil infiltrate (insets: intra-nuclear inclusion bodies with margination of nuclear chromatin, arrows). (**c**,**f**) Immunohistochemical detection of the N protein of RVFV.

We have previously reported that infectious RVFV was detected as early as one day post infection in sera of MBT mice and was undetectable in BALB/c mice at this timepoint. Furthermore, 3 days post infection, RVFV titers were shown to be three logs greater in MBT sera compared to BALB/c sera^11^. Since RVFV is known to induce acute-onset hepatitis in mice, concomitant with a rise in serum viral titer^6^, liver pathology of BALB/c and MBT strains was investigated. Serum levels of two liver enzymes, alanine aminotransferase (ALT) and aspartate transaminase (AST), were significantly elevated in MBT mice on day 3 post infection (median values 2844 and 3032 U/L, respectively), by comparison with uninfected controls (80 and 292 U/L, respectively; Mann-Whitney U test, p < 0.01 for both), suggesting an early onset of liver dysfunction. This phenomenon was not evident in BALB/c mice at day 3 post infection, with only mildy elevated ALT levels (510 U/L compared with 98 U/L in uninfected controls; Mann-Whitney U test, P < 0.01). However, in BALB/c mice, ALT and AST levels peaked on day 4 post infection (median values 9180 and 5280 U/L, respectively) and subsequently decreased, as reported previously^6^. Comparative histological analyses of livers from uninfected and day 3 infected BALB/c and MBT mice showed minimal to mild multifocal hepatitis, characterized by randomly distributed inflammatory foci containing mostly fragmented neutrophils in infected BALB/c and MBT mice. In these foci, necrotic and apoptotic hepatocytes could also be detected as well as cell debris. At the periphery of the lesions, acidophilic inclusion bodies could be identified in the nucleus of remaining hepatocytes (Fig. 2a,b,d and e). Immunochemistry with antibodies against the RVFV N protein revealed no differences in the distribution and profile of N-positive cells between BALB/c and MBT mice (Fig. 2c and f). The density of infected cells was indeed highly variable depending on liver sections, and on individual mice irrespective of the inbred strain. Altogether, survival, viral detection in blood, liver chemistry, and liver pathology indicate a neutrophil associated viral hepatitis with disparate survival time between the inbred strains, BALB/c and MBT.

### Higher viral antigen loads in the blood, spleen and liver of MBT mice

**Figure 3:**
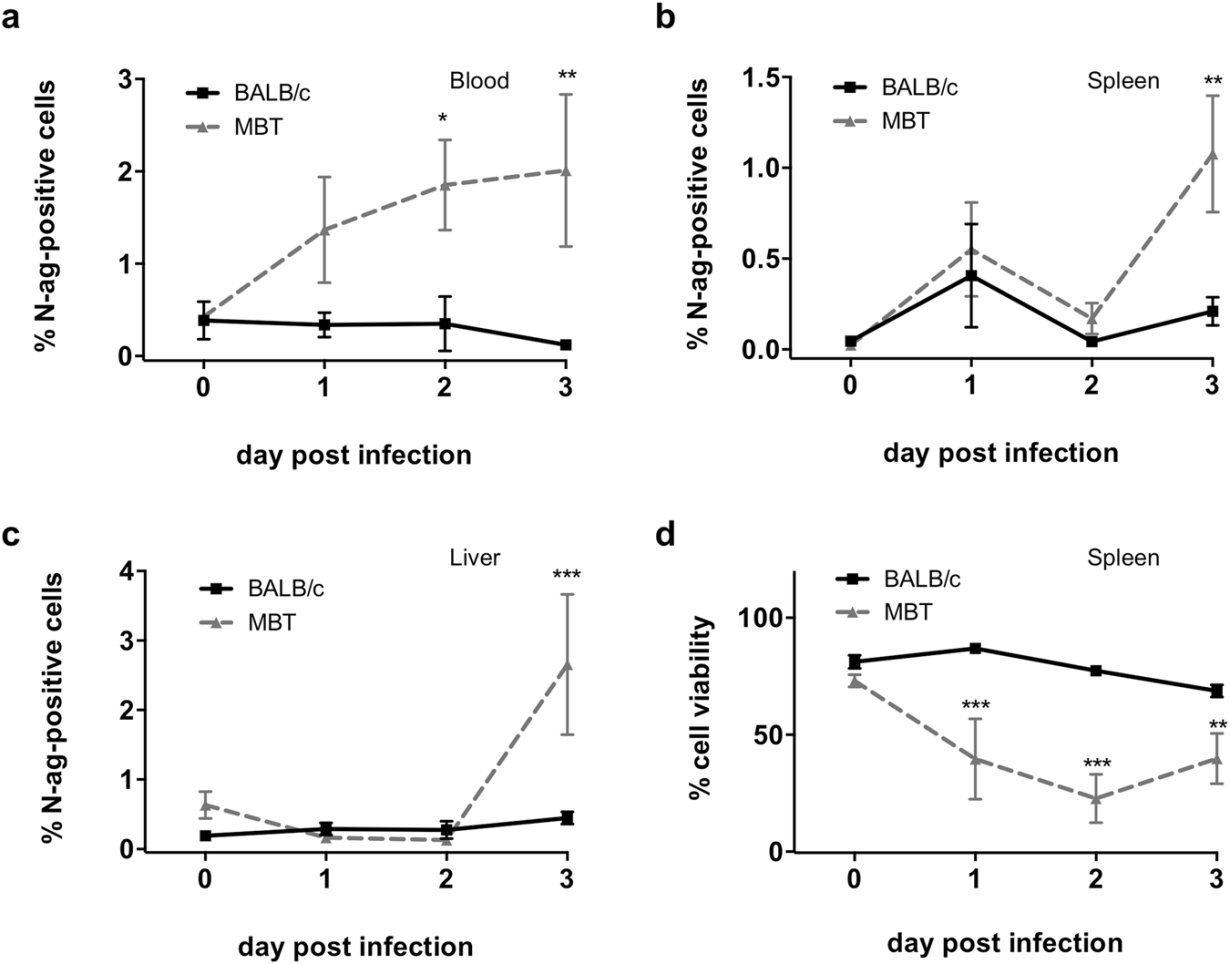
Higher viral antigen load correlates with higher cell death in the spleen of MBT mice. (**a**–**c)** Flow cytometric analysis for the N viral nucleoprotein (N-antigen, N-ag) within the blood (**a**), spleen (**b**), and liver (**c**) cells from BALB/c (n = 5/day) and MBT (n = 5/day) mice on days 1–3 post RVFV infection. **(d)** Percentages of viable cells in the spleen of RVFV-infected BALB/c and MBT mice using flow cytometry (n = 5 mice/strain/timepoint). Data are represented as mean ± SEM. Two-way ANOVA was used to test differences between means. *P < 0.05, **P < 0.01, ***P < 0.001.

We assessed levels of RVFV nucleoprotein (N) antigen in single-cell suspensions of the brain, blood, spleen, and liver from BALB/c and MBT mice on days 1–3 post RVFV infection using flow cytometric analysis. Virtually no viral antigen-positive cells were detected in the brain of BALB/c and MBT mice in that period (not shown). In the blood, the average percentages of viral antigen-positive cells were significantly higher on days 2 and 3 post infection in MBT mice compared to BALB/c mice (Fig. 3a). In the spleen and liver, higher levels of viral antigen-positive cells were detected at day 3 post infection in MBT mice when compared to BALB/c mice (Fig. 3b and c). The liver exhibited the highest percentages of viral antigen-positive cells at day 3 post infection (Fig. 3c, and Supplementary Fig. 1).

The primary lesion in the spleen of RVFV-infected mice is lymphocytolysis, as reported previously^6^. We assessed the spleen for markers of cell viability and apoptosis comparing mock-treated or RVFV-infected BALB/c and MBT mice. Results revealed a high percentage of viable cells on all days within the spleen of infected BALB/c mice, similar to levels in mock-treated BALB/c and MBT controls. By contrast, cell viability was greatly reduced in the spleen of infected MBT mice (Fig. 3d). This decreased cell viability was not due to caspase-3-dependent apoptosis (Supplementary Fig. 2). These data suggest increased cell death in the MBT spleen in association with increased viral antigen load.

### Differential distribution of innate immune cells in BALB/c and MBT mice

**Figure 4:**
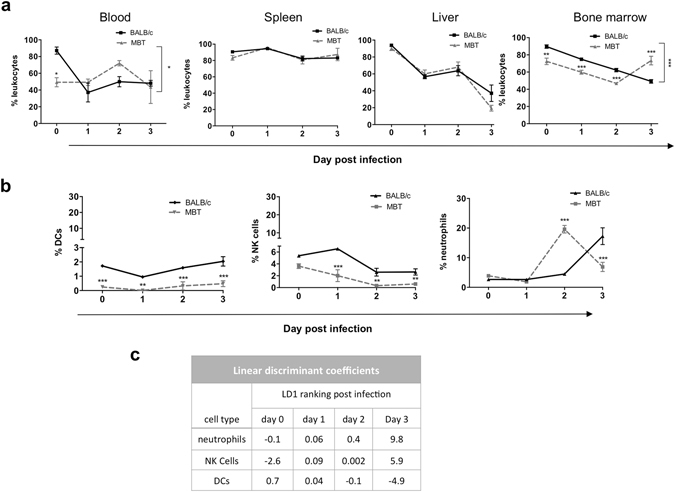
Changes in the percentages of innate immune cells in RVFV-infected BALB/c and MBT mice. **(a**) A comprehensive flow cytometric profiling of leukocytes defined as positive for CD45 in the blood, spleen, liver and bone marrow of RVFV-infected BALB/c and MBT mice on days 0–3 post infection. (**b**) Percentages of key innate immune cell populations, DCs (CD45+, CD11c+), natural killer (NK) cells (CD45+, CD335+), and neutrophils (CD45+, Ly6C/Ly6G+), after infection with RVFV in the spleen of BALB/c and MBT mice. **(c)** Linear discriminant (LDA) analyses ranks neutrophils, as having the greatest impact on the dissimilarity between BALB/c and MBT spleen at day 3 post infection. Cytometry experiments were performed twice for each time point (n = 5 mice/strain/timepoint). Representative experiments are displayed. Data are represented as mean ± SEM. Two-way ANOVA was used to test differences between means. *P values < 0.05, **P < 0.01, and ***P < 0.001.

To assess the impact of RVFV infection on innate immune cell frequency and subset distribution, a comprehensive flow cytometric profiling of key innate immune cells in the blood, spleen, liver and bone marrow of mock- and RVFV-infected BALB/c and MBT mice was performed on days 0–3. In BALB/c mice, results revealed a drop in circulating CD45-positive leukocytes starting with the infection (Fig. 4a). A steady decrease in the leukocyte levels was also registered in the liver and bone marrow post infection. The counts of the leukocytes in the liver and bone marrow of BALB/c mice were at their lowest levels at day 3 after infection (Fig. 4a). The overall leukocyte levels in the spleen did not change during the investigated period. In MBT mice, the leukocyte levels in the blood and bone marrow were already lower before infection compared to non-infected BALB/c mice (Fig. 4a). Interestingly, circulating MBT leukocytes and leukocytes in the spleen and liver were maintained at comparable levels as in BALB/c mice, despite lower levels of leukocytes originating from MBT bone marrow at days 0–2 post infection. Data from the bone marrow also suggests that the systemic deficit in MBT leukocytes was remedied at day 3 post infection when MBT leukocyte levels surpassed that of BALB/c (Fig. 4a).

To identify the key cell subsets of leukocytes affected after infection, we narrowed the experimental focus to evaluate organs with high viral load, the spleen and liver, and surveyed numerous cells at the stage when the average MBT mouse was within 24 hours of succumbing to RVFV-induced fatality, at day 3 post infection. We profiled for dendritic cells (DCs), natural killer (NK) cells, neutrophils, macrophages, and B-cells. We also evaluated for the potential of early adaptive cell response by assessing surface CD4 and CD8 T cell expression (Supplementary Fig. 3). Experimental analysis identified three cell subsets, DCs, NK cells, and neutrophils in the spleen, as significantly lower in MBT mice when compared to the more resistant BALB/c mice (Mann-Whitney U test, P < 0.05) (Fig. 4b, data in liver not shown). These cell types subsequently became the focus for our comprehensive study.

Informative data were obtained in the spleen of MBT mice where DC levels, already low before infection when compared with BALB/c mice, decreased further at day 1 post infection (Fig. 4b). Similarly, NK cell levels dropped on days 1 and 2 post infection in MBT mice to reach very low values (Fig. 4b). The most striking observation was the rise in neutrophils at day 2 post infection, which occurred earlier than in BALB/c mice, followed by a sudden drop at day 3 post infection which did not happen in BALB/c mice (Fig. 4b). To best recognize cell subtype contribution differentiating BALB/c and MBT strains, principal component (PCA) and linear discriminant (LDA) analyses were performed on innate cell profiles in the spleen at days 0–3 post infection. LDA analysis ranked neutrophils as having most impact starting on day 2 and persisting until day 3 post infection on the dissimilarity between infected BALB/c and MBT mice (Fig. 4c). Collectively, flow cytometric profiling of key innate immune cells in MBT mice argues for abrupt and large increases in splenic neutrophils at day 2 post infection and in bone marrow-derived leukocytes on day 3 post infection. These increases were not observed in BALB/c mice. During the investigated period, DCs remained at very low levels in MBT spleen, while splenic NK cells even reduced to attain lower levels than in BALB/c.

### Exhaustion of neutrophils in the blood and bone marrow of MBT mice

**Figure 5:**
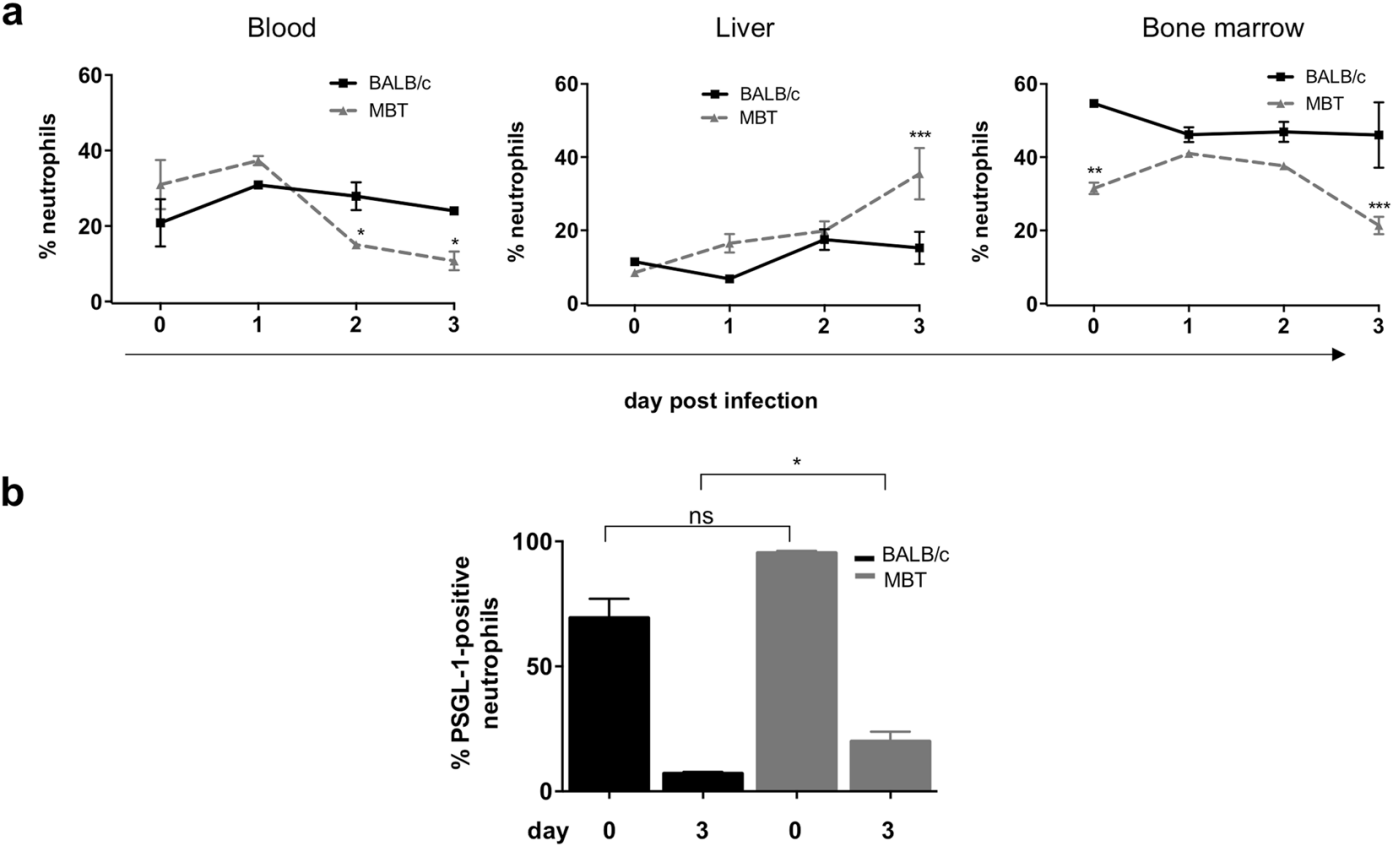
Faster recruitment of neutrophils, and higher PSGL-1 levels in neutrophils at day 3 post infection in MBT mice. (**a**) Flow cytometric profile of neutrophil (CD45+, Ly6C/Ly6G+) populations in the blood, liver, and bone marrow of RVFV-infected BALB/c and MBT mice on days 0–3 post infection. Two-way ANOVA was used to test differences between means **(b)** Percentages of PSGL-1-positive circulating neutrophils at day 3 post infection. Cytometry experiments were performed twice (n = 5 mice/strain/timepoint, unless otherwise indicated). Data are represented as mean ± SEM. Mann- Whitney U test was used to compare differences between means. *P < 0.05, **P < 0.01, and ***P < 0.001.

Flow cytometric profiling showed that circulating neutrophil levels were similar in mock-infected and on day 1 post infection in both mouse strains, but they became lower on days 2 and 3 post infection in MBT strain when compared to BALB/c strain (Fig. 5a). In the BALB/c bone marrow, neutrophil levels declined on day 1 post infection and remained at steady levels afterwards. By contrast, an increase in neutrophil levels was observed at day 1 post infection in MBT bone marrow followed by a progressive decline (Fig. 5a). In the liver, a modest increase in neutrophil level was observed in BALB/c mice on day 2 post infection. By contrast, a steady and steep increase in neutrophil levels was observed in the MBT liver (Fig. 5a). Neutrophil levels dropped in the MBT blood and bone marrow even more severely between days 1 and 3 post infection compared to BALB/c mice (Fig. 5a). These results suggest that, in MBT mice, neutrophils accumulate progressively in the liver where the viral antigen load is the highest at day 3 post infection, at the expense of the blood, spleen, and bone marrow.

We investigated neutrophil activation by measurement of intracellular IFN-γ in the blood, spleen and liver at day 3 post infection. IFN-γ expression trended lower in circulating and splenic neutrophils in MBT mice compared with BALB/c mice, whereas IFN-γ levels in liver neutrophils trended higher in MBT mice compared with BALB/c mice (Supplementary Fig. 4).

Another marker of neutrophil activation we exploited is P-selectin glycoprotein ligand-1 (PSGL-1). Its function is vital during leukocyte recruitment into tissues and its regulation is integral during acute inflammation. Slow rolling of leukocytes on the endothelial cells lining venules of the tissues is the earliest observable event of this physiological reaction. The initial tethering and rolling of circulating leukocytes is mediated by PSGL-1 that binds to P-selectin expressed by endothelial cells^24^. In addition to its tethering function by endothelial cells, PSGL-1 can nucleate leukocyte-leukocyte interactions by binding to L-selectin also expressed on the surface of circulating leukocytes, thus enabling secondary leukocyte capture^25, 26^. We measured PSGL-1 levels on circulating neutrophils during infection in BALB/c and MBT mice. At day 3 post infection, higher percentages of PSGL-1-positive neutrophils were observed in MBT compared to BALB/c blood (Fig. 5b), suggesting enhanced tethering and rolling ability of MBT neutrophils to site of inflammation at that time. This conclusion is consistent with the increased levels of neutrophils in the liver of MBT mice compared with BALB/c mice at day 3 post infection (Fig. 5a).

Neutrophil recruitment was studied after injection of thioglycollate into the peritoneal cavity of BALB/c and MBT mice. Similar numbers of neutrophils were observed in the thioglycollate-stimulated peritoneal exudates of BALB/c and MBT mice. This suggests that MBT neutrophils do not have higher capacity to be recruited to a site of inflammation (Supplementary Fig. 5).

Taken together these data indicate that, after a faster response to the infection, MBT mice sustained lower levels of circulating neutrophils than BALB/c mice. To understand if the level of circulating neutrophils is critical during RVF disease, we used C57BL/6 mice that carry either null (*Gfi1* ^*GFP*^) or hypomorphic (*Gfi1* ^*Gen*^) alleles at the *Gfi1* locus. Homozygous *Gfi1* ^*GFP/GFP*^ mice show a severe neutropenia and additional defects in the B- and T-cell lineages and in the hematopoietic stem cell fraction while heterozygous *Gfi1* ^+*/GFP*^ littermates are indistinguishable from wild type mice^27^. Homozygous *Gfi1* ^*Gen/Gen*^ mice are almost deprived of mature neutrophils, but they exhibit milder defects on hematopoietic precursors and on the development of lymphoid cells^28^. Survival curves of *Gfi1* ^*GFP/GFP*^ and *Gfi1* ^+*/GFP*^ mice on the one hand, and *Gfi1* ^*Gen/Gen*^ and *Gfi1* ^+/+^ mice on the other hand did not differ significantly after infection (Supplementary Fig. 6a and b, respectively). These results suggest that neutrophils are not critical during RVF disease.

### Expression of type I interferons and their receptor in susceptible MBT mice

**Figure 6:**
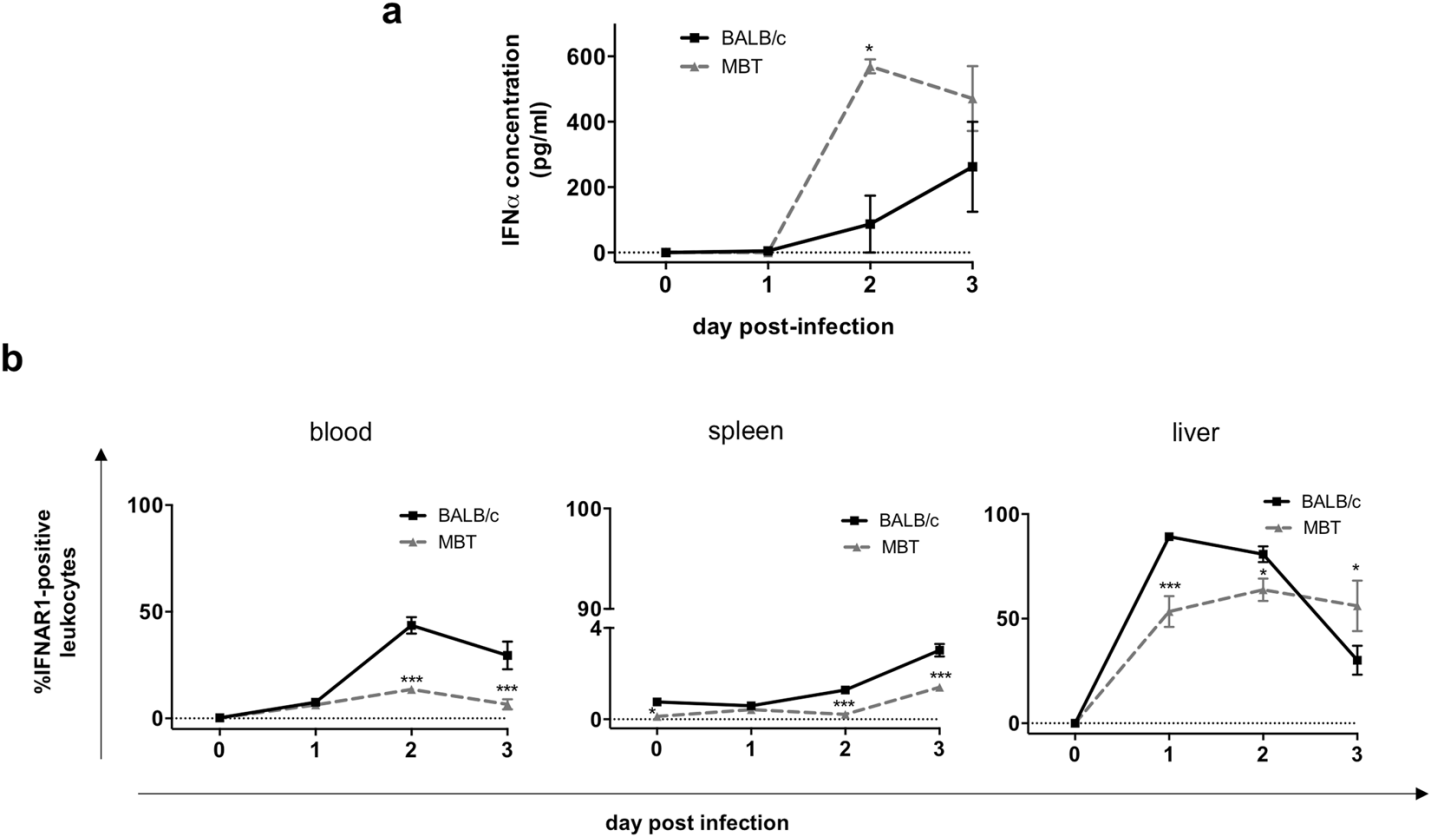
Greater IFN-α expression, but weaker IFNAR1 expression in MBT mice. **(a)** Serum IFN-α concentrations in BALB/c and MBT mice on days 0–3 post infection (n = 8 per strain, infected, n = 3 per strain, sham-infected). **(b)** Percentages of total IFNAR1-positive leukocytes in the blood, spleen, and liver of BALB/c and MBT mice as measured by flow cytometry. Cytometry experiments were performed twice (n = 5 mice/strain/timepoint). Representative experiments are displayed. Data are represented as mean ± SEM. Two-way ANOVA was used to test differences between means. *P values < 0.05, **P < 0.01, and ***P < 0.001.

Increased IFN-α/β response is a key mechanism of innate immunity to protect the host against invading viruses^29^. We assayed IFN-α/β in the serum of mock- and RVFV-infected BALB/c and MBT mice between days 1 and 3 post infection. NSs, the major virulence factor of RVFV is known to inhibit the synthesis of *Ifnb1* gene encoding IFN-β^30^. Accordingly, IFN-β remained below detection level in sera from infected BALB/c and MBT mice. However, significant concentrations of the related interferon-α were measured in BALB/c and MBT mice at day 2 post infection, with a stronger IFN-α expression in MBT mice (569 ± 21 pg/ml) compared to BALB/c mice (87 ± 87 pg/ml; two-way ANOVA, P < 0.05) (Fig. 6a). No differences between strains were seen at day 3 post infection (262 ± 137 pg/ml and 471 ± 99 pg/m in BALB/c and MBT mice, respectively; two-way ANOVA, P = 0.31) (Fig. 6a). Thus, MBT mice are able to induce rapidly a much stronger IFN-α response than BALB/c mice.

The levels of IFN-α and -β receptor 1 (IFNAR1) on the cell’s surface determine the sensitivity of cells to type I IFNs and control the magnitude and duration of effect elicited by these cytokines^14, 31^. We investigated if this viral trigger for innate immune defense was functional in MBT mice via measurement of IFNAR1 cell surface expression. Following infection with RVFV, the percentages of IFNAR1-positive leukocytes increased dramatically in the blood, spleen and liver of BALB/c mice (Fig. 6b). By contrast, the IFNAR1-positive leukocyte increases were less pronounced in the blood and spleen of MBT mice. In the MBT liver, the increase was also weaker compared to the BALB/c liver, but by day 3 post infection IFNAR1-positive leukocyte levels surpassed levels found in BALB/c liver (Fig. 6b).

Collectively, these data indicate that RVFV infection induced an increase in the percentage of leukocytes expressing type I IFN receptor 1 in BALB/c mice, potentially strengthening the cellular response to type I IFNs in the course of infection. By contrast, a smaller percentage of leukocytes expressed IFNAR1 in MBT mice, suggesting a lower ability to trigger a response to type I IFNs compared to BALB/c mice.

### Delayed activation of circulating leukocytes in MBT mice

To further evaluate the activation of immune cells in MBT mice and their subsequent capacity to mobilize during RVFV infection, we examined IFN-γ production in circulating leukocytes and their surface expression of PSGL-1.

**Figure 7:**
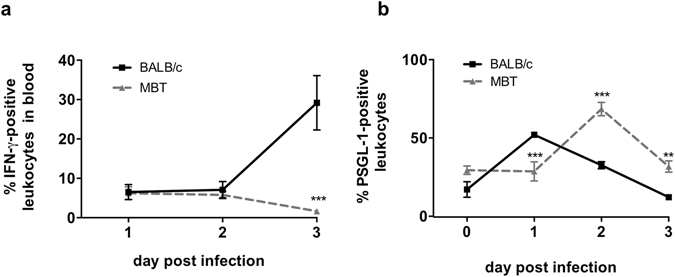
Delayed activation of circulating leukocytes in MBT mice. (**a)** Percentages of circulating leukocytes expressing IFN-γ in BALB/c and MBT mice on days 1–3 post infection as evaluated by intracellular flow cytometry. **(b)** Percentages of PSGL-1-positive circulating leukocytes in BALB/c and MBT mice on days 0–3 post infection. Cytometry experiments were performed twice (n = 5 mice/strain/timepoint). Data are represented as mean ± SEM. Representative experiments are displayed. Two-way ANOVA was used to test differences between means. *P < 0.05, **P < 0.01, and ***P < 0.001.

Activation of leukocytes in the blood of BALB/c and MBT mice was evaluated by the production of IFN-γ at each day after infection. The IFN-γ-positive leukocyte population increased at day 3 post infection in BALB/c blood, indicating that circulating leukocytes were activated at this stage. By contrast, the population of IFN-γ-positive leukocytes was not observed in the bloodstream of MBT mice (Fig. 7a).

The initial tethering and rolling of circulating leukocytes mediated by PSGL-1 precedes firm attachment needed for leukocyte arrest before extravasation. Firm attachment has been suggested to be facilitated by leukocyte activation and PSGL-1 downregulation^32^. Upon activation by a variety of stimuli, the expression of PSGL-1 on the surface of circulating leukocytes decreases^32–34^. We assayed leukocytes that expressed PSGL-1 on days 0 to 3 after infection in BALB/c and MBT blood. Figure 7b shows that, at day 1 post infection, PSGL-1-positive leukocyte levels were higher in BALB/c than in MBT blood suggesting greater tethering ability of BALB/c leukocytes at this early stage. Interestingly from that moment onwards, PSGL-1-positive leukocyte levels declined gradually in BALB/c blood suggesting that activation of circulating leukocytes started on day 1 post infection in BALB/c mice. By contrast, in MBT blood, the highest levels of PSGL-1-positive leukocytes were observed on day 2 post infection, that was also the time when the levels of PSGL-1-positive leukocytes began to decrease. These data suggest delayed activation of circulating leukocytes in MBT mice. These results suggest that RVFV induces earlier activation of circulating leukocytes in BALB/c mice.

## Discussion

MBT mice survive after infection with West Nile virus or influenza A virus in conditions where BALB/c mice die. However, after inoculation with RVFV, MBT mice died at a median of 3 days, with histological and biochemical signs of acute hepatitis, while BALB/c mice survived up to a week longer. An increase in splenic neutrophils on day 2 post infection suggests an earlier inflammatory response in MBT mice. This is in line with the higher IFN-α concentration in MBT serum on day 2 post infection. However, despite the protection normally conferred by IFN-α, MBT mice had a rapid rise in viral antigen load, suffered from hepatitis, and eventually died. In fact, MBT mice display several immune-related defects. Notable amongst those are the levels of leukocytes expressing IFNAR1 which were lower in the blood, spleen, and liver of MBT mice than in BALB/c mice. IFNAR1 is crucial in engaging innate cell machinery to restrict viral spread, and in expanding sufficient leukocyte numbers to tackle intracellular infection. Signaling through receptor type I IFNs induces hundreds of genes to establish an antiviral state^35^. Although RVFV-infected MBT mice produced serum IFN-α, their lower percentage of IFNAR1-positive leukocytes compared to BALB/c mice suggests a weakened type I IFN response in MBT mice. Lack of IFNAR1 expression in subsets of myeloid cells has been shown to enhance West Nile virus infection and tissue injury in visceral organs, including the liver^36^. Thus, decreased percentage of leukocytes expressing IFNAR1 likely contributes to the susceptible MBT phenotype.

Recruitment of circulating leukocytes from blood to infected tissues is a crucial component of host defense mechanisms. P-selectin glycoprotein ligand-1 (PSGL-1) is the primary ligand for P-selectin. PSGL-1 is constitutively expressed on the surface of circulating leukocytes while P-selectin is expressed by endothelial cells^24, 37^. Activation of circulating leukocytes is associated with decreased surface expression of PSGL-1. This down-regulation decreases leukocyte adhesion to P-selectin expressed by the vascular endothelium thus favoring the transition from weak selectin-mediated interaction to firm attachment via the β2 integrins and ICAM-1^32, 38^. We have shown that, following infection, circulating leukocytes exhibited a delayed down-regulation of PSGL-1 in MBT mice compared to BALB/c mice.

IFN-γ is a pro-inflammatory mediator produced in response to intracellular pathogens^39^. IFN-γ has been shown to reduce viremia, thus eliminating clinical signs in RVFV-infected rhesus monkeys^40^. IFN-γ also serves as an activation marker. We have observed that the levels of circulating leukocytes that produced IFN-γ 3 days after infection were low in MBT mice compared to BALB/c mice. These observations can be explained in several ways. First, circulating MBT leukocytes failed to express IFN-γ as a result of poorly efficient or delayed activation despite a higher viral antigen load in the blood. This hypothesis is supported by the delayed down-regulation of PSGL-1 seen in circulating MBT leukocytes. Second, in MBT mice, activated (IFN-γ-positive) leukocytes migrated to the liver where the viral antigen load was the highest, and were therefore lacking in the blood. Both mechanisms can contribute independently to the weak levels of IFN-γ-positive leukocytes in the blood of MBT mice.

LDA analysis on immune cell subsets ranked neutrophil level as the major variable discriminating infected BALB/c and MBT mice at day 3 post infection. Circulating neutrophils were indeed at low levels in RVFV-infected MBT mice, and the capacity to replenish this population was reduced compared to BALB/c mice. It has previously been shown that neutropenia can contribute to susceptibility to viral infections, for example, in the case of Simian Immunodeficiency Virus (SIV) infections of nonhuman primates^41^ (reviewed in ref. 42). However, we have shown that C57BL/6 mice homozygous for either *Gfi1* ^*Gen*^ or *Gfi* ^*GFP*^, two alleles associated with severe neutropenia, were not more susceptible than wild type C57BL/6 mice, suggesting that low number of neutrophils does not worsen RVF disease. Therefore, it is likely that the neutropenia observed in infected MBT mice is the result of the pathogenesis associated with RVFV infection rather than a major causal component of the susceptibility. A second non-mutually exclusive possibility is that neutropenia impinges negatively on health status in the MBT genetic background only.

Altogether, the results presented in this paper show that despite an earlier inflammatory response, MBT susceptible mice are unable to mount and maintain an adequate response against RVFV. Our study thus provides novel evidence for understanding the links and markers of pathogenesis of variable disease progression between infected individuals, and the analysis of the complex genetic component of susceptibility to RVF disease.

## Materials and Methods

### Mice, virus, infection and tissue harvest

Experiments on live mice were conducted according to the French and European regulations on care and protection of laboratory animals (EC Directive 2010/63/UE and French Law 2013-118 issued on February 1, 2013). Experimental protocols were approved by the Animal Ethics Committee of Institut Pasteur (N° 89). All experiments that involved virulent RVFV were performed in the biosafety level 3 (BSL3) facilities of the Institut Pasteur, and carried out in compliance with the recommendations of the Institut Pasteur Biosafety Committee (N° 14.320).

BALB/cByJ, MBT/Pas, PWK/Pas, SEG/Pas, 129/SvPas, C57BL/6 J, *Gfi1* ^*GFP/GFP*^ and *Gfi1* ^+*/GFP*^ C57BL/6 mice were bred and maintained at Institut Pasteur^23, 27, 43–45^. *Gfi1* ^*Gen/Gen*^ and *Gfi1* ^+/+^ C57BL/6 controls were obtained from *Centre d’Immunologie de Marseille-Luminy* (Marseille, France)^28^. All studies used RVFV strain ZH548, obtained from *Centre National de Référence des Fièvres Hémorragiques Virales* (Institut Pasteur, Lyon, France)^46, 47^. Because sex influences the severity of infection^12^, only male mice were used. Approximately 10^2^ PFU of ZH548 RVFV were injected intraperitoneally into 9–14 week-old BALB/c and MBT mice^11^. Infected mice were monitored daily for clinical symptoms. Clinical scores included no symptoms, mild lethargy (slow gait), paralysis or neurological displays (spinning and/or head-tilting), and severe lethargy (a combination of slow gait and decreased response time to stimulus). Mice were provided with water and wet chow when paralyzed, and euthanized when moribund. Mice that survived until day 15 post infection were also euthanized. Blood was taken by cardiac puncture, while femur bone marrow, brain, liver and spleen were removed by manual dissection. Bone marrow from the left and right femur was collected by quick centrifugation of bone in a perforated 0.5 ml Eppendorf tube placed into a 1.5 ml Eppendorf tube supplied with 200 μl of Dulbeccos’s Phosphate Buffered Saline (PBS). Entire organs were processed for flow cytometric analysis and histology.

### IFN-α and IFN-β Measurements

Blood was collected by cardiac puncture from mock- (n = 3 per strain) and RVFV-infected (n = 8 per strain) MBT and BALB/c mice. IFN-α and IFN-β were assayed using the mouse IFN-α and IFN-β ELISA kits (PBL Biomedical Laboratories, Piscataway, NJ). The kits’ lower limits of quantification were 12.5 and 15.6 pg/ml for IFN-α and IFN-β, respectively.

### Liver Enzymes Measurement

Blood was collected from mock- (n = 3 per strain) and RVFV-infected (n = 9 per strain) MBT and BALB/c mice by cardiac puncture. Sera were analyzed on a VetTest chemistry analyzer (IDEXX laboratories, Westbrook, ME) to determine alanine aminotransferase (ALT) and aspartate transaminase (AST) levels.

### Histopathology

For histological analysis, whole liver (n = 7 per strain) was fixed for one week in 10% neutral-buffered formalin, then immersed for 24 hrs in 70% ethanol. Samples from four representative areas of each lobe were embedded in paraffin, and 5 μm-thick sections were cut and stained with hematoxylin and eosin (HE). Slides were visualized with the Histofine Simple Stain MAX-PO kit (Nichorei Biosciences Inc., Tokyo, Japan). The histological characterization of liver lesions was completed by an immunohistochemical detection of the RVFV using mouse antibodies against the N protein of RVFV (dilution 1:100). Sections were analyzed in a blind study on coded slides to evaluate the histological architecture and to identify presence of virus and neutrophils.

### Flow Cytometry Analysis

Mice (n = 5 per strain/3 independent experiments) were transcardially perfused with PBS until liver blanched. Entire organs were harvested in Hank’s Balanced Salt Solution (HBSS). Single-cell suspensions were prepared after mechanical disruption with the gentleMACS Dissociator (Miltenyi Biotec, San Diego, CA). Liver was exposed to a first dissociation cycle, and incubated at 37 °C for 20 min with 100 IU/ml collagenase IV (Worthington, Lakewood, NJ) in HBSS before a second cell disruption cycle. Cell suspensions from liver and brain were filtered through a 40 μM cell strainer, and purified by Ficoll-Paque density gradient (GE Healthcare Life Sciences, Marlborough, MA). Cell suspensions were treated with red blood cell lysis buffer (Miltenyi Biotec SAS), diluted to 1–8 million cells/ml, incubated with the mouse 2.4G2 FcyII blocker (BD Biosciences, San Jose, CA), stained for surface markers over a 1 hr incubation period, fixed in 4% PFA solution, and resuspended in albumin 2% in PBS (PAN-Biotech, Aidenbach, Germany). Antibodies used for staining are listed in Supplementary Table 1. For intracellular staining, cells were treated with a permeabilization buffer (eBioscience, San Diego, CA) before a 90 min staining incubation. Leukocytes were classified as CD45+; neutrophils were identified as CD45+ Ly6C/6 G+; NK cells as CD45+ CD335+; macrophages as CD4+ CD11b+ CD11c−; B-cells as CD45+ CD19+; cytotoxic T-cells as CD45+ CD3e+ CD8+; helper T-cells as CD45+ CD3e+ CD4+; and conventional dendritic cells (DCs) as CD45+, CD1b+, CD11c+. PSGL-1 and IFNAR1 were assessed by surface staining antibodies. Caspase-3 and IFN-γ were measured post surface staining by the intracellular staining protocol. Fluorescence was measured using a four-laser LSRII flow cytometer (BD Biosciences). For each samples a total number of at least 200,000 cells was analyzed. Dead cells were visualized using eFluor 780 (eBioscience) (For example of typical gating scheme see Supplementary Fig. 7). When cell populations did not stain to generate two clearly distinct populations, gates were drawn to isolate the majority of all positive cell populations, and the gates were applied to experimental and control samples alike. Gating was determined by both single-stained control, and fluorescence minus one control. Data analysis was performed using FlowJo software version 10 (Ashland, OR).

### Thioglycollate Neutrophil Migration

Peritonitis was induced by intraperitoneal injection of 1 ml sterile thioglycollate 3% in PBS (Sigma-Aldrich, St. Louis, MO) into naive BALB/c and MBT mice (n = 5 per strain/2 independent experiments). In parallel BALB/c and MBT mice were injected 1 ml PBS, as a control. Peritoneal cavities were washed with 2 × 5 ml of ice-cold sterile PBS, and the cells were subsequently centrifuged and resuspended for flow cytometry analysis.

### Statistical Analysis

Measurements were carried out on individual mice, and the mean and standard error of mean (SEM) were calculated for each mouse strain. Mantel-Cox’s Logrank test was applied to assess survival curve differences. Two-way ANOVA was used to compare percentages of innate immune cells, cell viability, N-ag-, IFN-γ-positive cells, and IFN-α levels during infection. Mann-Whitney U test was used to analyze levels of liver enzymes. PCA and LDA analyses were performed with R software version 3.0.0^48^. All other analyses were performed with Prism 6.02 (GraphPad, San Diego, CA). Significance was considered for P values < 0.05 (*), but included P values < 0.01 (**), and < 0.001 (***) as indicated.

### Data Availability

All data generated or analyzed during this study are included in this published article (and its Supplementary Information files).

## Electronic supplementary material

Supplementary information
