# Supplementary material for: Innate Immune Basis for Rift Valley Fever Susceptibility in Mouse Models
Source: Sci Rep. 2017 Aug 2;7:7096. doi: 10.1038/s41598-017-07543-8 (PMC5541133; doi:10.1038/s41598-017-07543-8)
Supplement: Supplementary file 1 — Supplementary information [file 41598_2017_7543_MOESM1_ESM.pdf]

SUPPLEMENTARY INFORMATION

Innate Immune Basis for Rift Valley Fever Susceptibility in Mouse Models

Rashida Lathan, Dominique Simon-Chazottes, Grégory Jouvion, Ophélie Godon, Marie Malissen, Marie Flamand, Pierre Bruhns & Jean-Jacques Panthier

Supporting information Captions

Supplementary Figure 1. The liver exhibits the highest percentages of cells positive for the RVFV N antigen (N-ag). Percentages of N-ag-positive cells in the blood (n=8), spleen (n=26), and liver (n=15) cells of BALB/c mice at day 3 post infection. Data are represented as mean ± SEM. Groups were analyzed with two-way ANOVA. **p <0.01, n.s. means non-significant.

Supplementary Figure 2. The decreased cell viability of cells in the spleen of MBT compared to BALB/c mice is not due to caspase-3- dependent apoptosis. Percentages of caspase-3-positive cells in the spleen of RVFV-infected BALB/c and MBT mice at day 3 post infection. Cytometry experiments were performed twice, n=5 mice/strain/timepoint. Data are represented as mean ± SEM. Groups were analyzed with two-way ANOVA. n.s. means non-significant.

Supplementary Figure 3. Differences were not found between other innate immune cells subsets in RVFV-infected BALB/c and MBT mice. Flow cytometric profiling of CD4 T-cells (CD45+, CD3+, CD4+), CD8 T-cells (CD45+, CD3+, CD8+), macrophages (CD45+, CD11b+), and B-cells (CD45+, CD19+) in the spleen (**a**) and liver (**b**) of RVFV-infected BALB/c and MBT mice on day 3 post infection. Cytometry experiments were performed 2 times (n=3-5 mice/strain). Data are represented as mean ± SEM. A Mann-Whitney U test was used to determine differences between means, n.s. means non-significant.

Supplementary Figure 4. Characterization of IFN-γ expression in neutrophils. Percentages of IFN-γ-positive neutrophils in the blood, spleen and liver of BALB/c and MBT mice at day 3 post infection as determined by intracellular staining in flow cytometry. Experiments were performed 2 times (n=5 mice/strain). Data are

represented as mean ± SEM. Mann-Whitney U test was used to determine differences between means, n.s. means non-significant.

Supplementary Figure 5. Recruitment of neutrophils following sterile inflammation. Absolute number of neutrophils in the peritoneal space of BALB/c and MBT mice 48 hours after stimulation with thioglycollate. Experiments were performed 2 times (n=5 mice/strain). Data are represented as mean ± SEM. Mann-Whitney U test was used to determine differences between means, n.s. means non-significant.

Supplementary Figure 6. Constitutive neutropenia has not effect on survival of infected mutant and wild type C57BL/6 mice. (a) Survival curves of $Gfi1^{GFP/GFP}$ and $Gfi1^{+/GFP}$ C57BL/6 mice following infection with RVFV. **(b)** Survival curves of *Genista* C57BL/6 (n=6), wild-type C57BL/6 (n=6), BALB/c (n=30), and MBT (n=27) mice following infection with RVFV. Mantel-Cox's Logrank test was performed to assess survival curve differences.

Supplementary Figure 7. Typical gating scheme used to identify immune cell subpopulations. Example of neutrophils (Ly6CG+) that were positive for PSGL-1 (CD162), taken from a BALB/c mouse at day 1 post infection. Each sample consisted of at least 200,000 viable cells (FSC × SSC gated and viability e780 negative). All analyzed leukocytes were CD45+. Gating was determined by both single-stained control, and fluorescence minus one control.

Supplementary Table 1. Antibodies used for staining.

Supplementary Figure 1

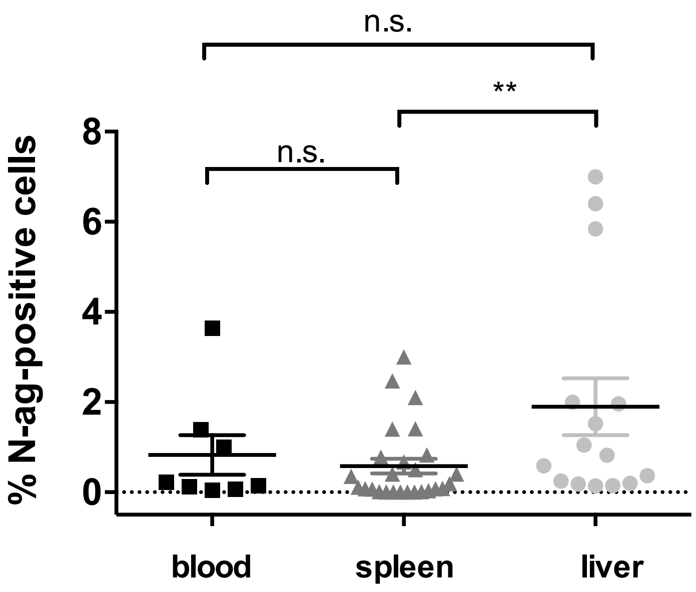

The liver exhibits the highest percentages of cells positive for the RVFV N antigen (N-ag).

Supplementary Figure 2

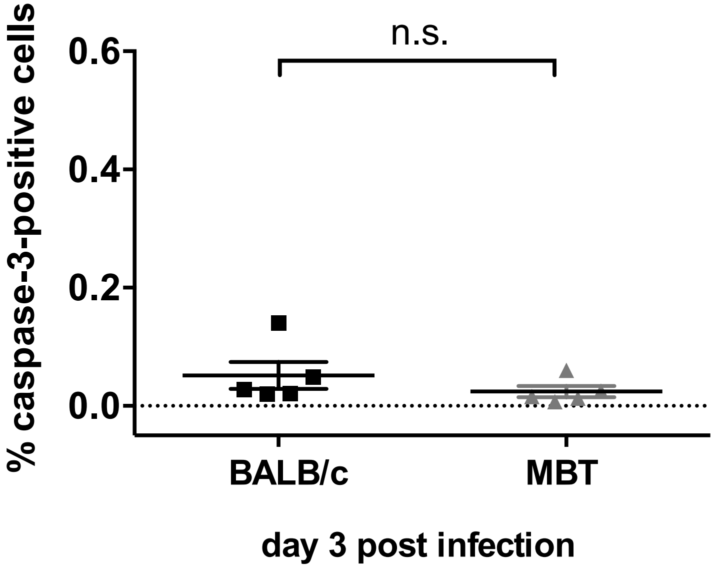

The decreased cell viability of cells in the spleen of MBT compared to BALB/c mice is not due to caspase-3- dependent apoptosis.

Supplementary Figure 3

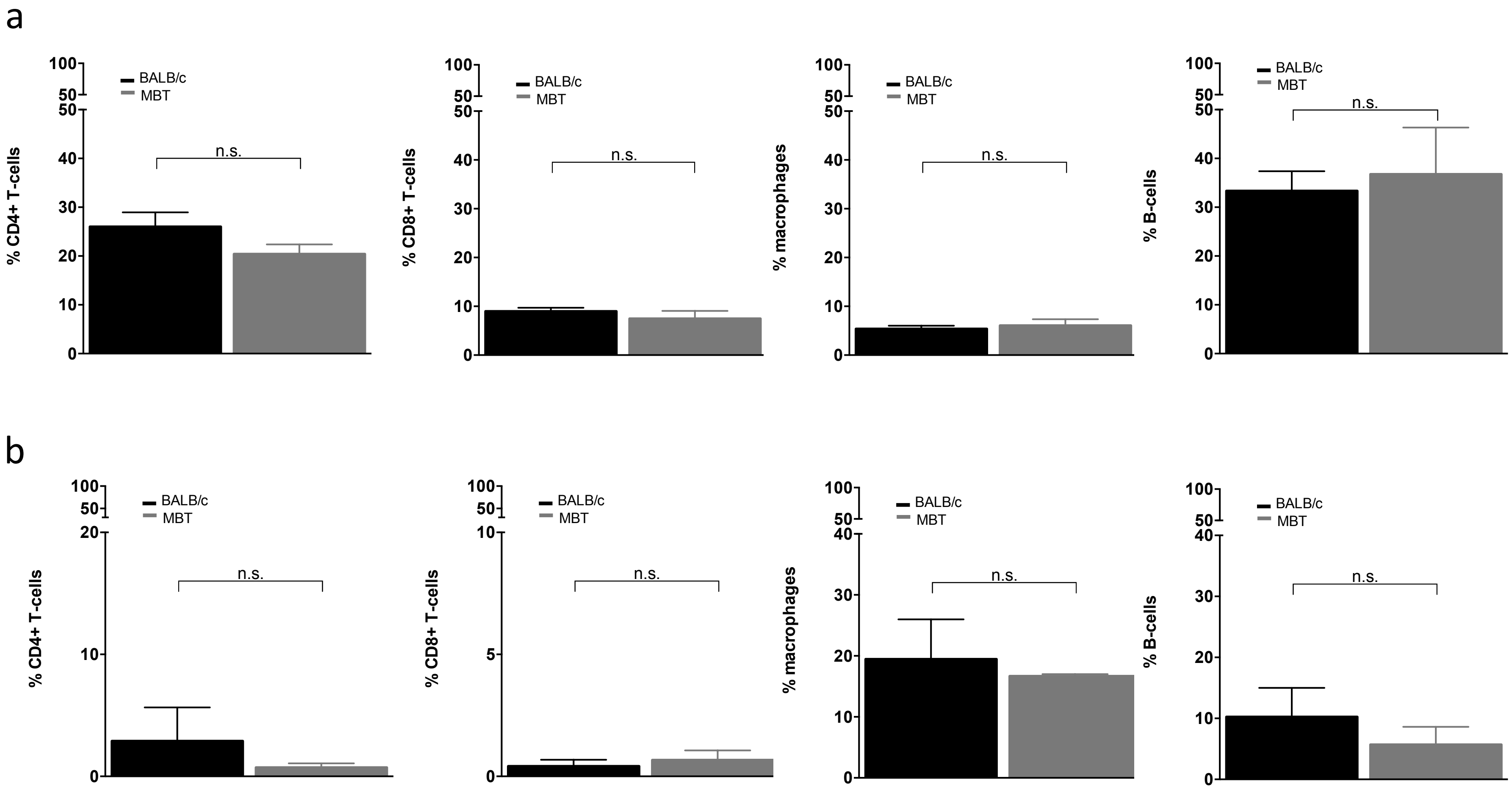

Differences were not found between other innate immune cells subsets in RVFV-infected BALB/c and MBT mice.

Supplementary Figure 4

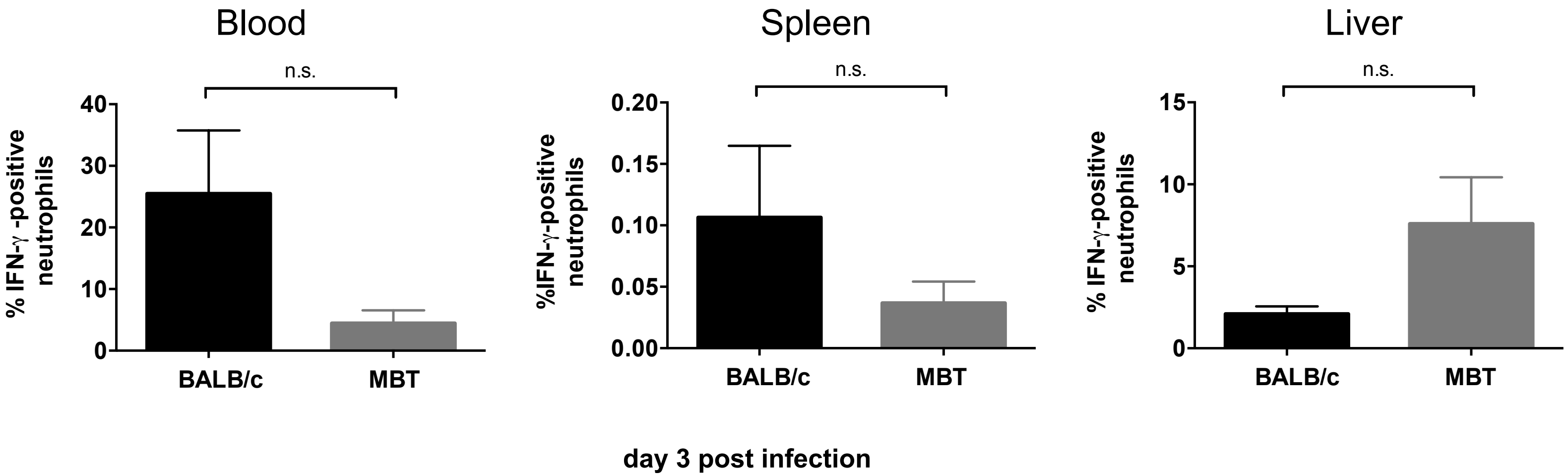

Characterization of IFN-γ expression in neutrophils.

Supplementary Figure 5

Recruitment of neutrophils following sterile inflammation.

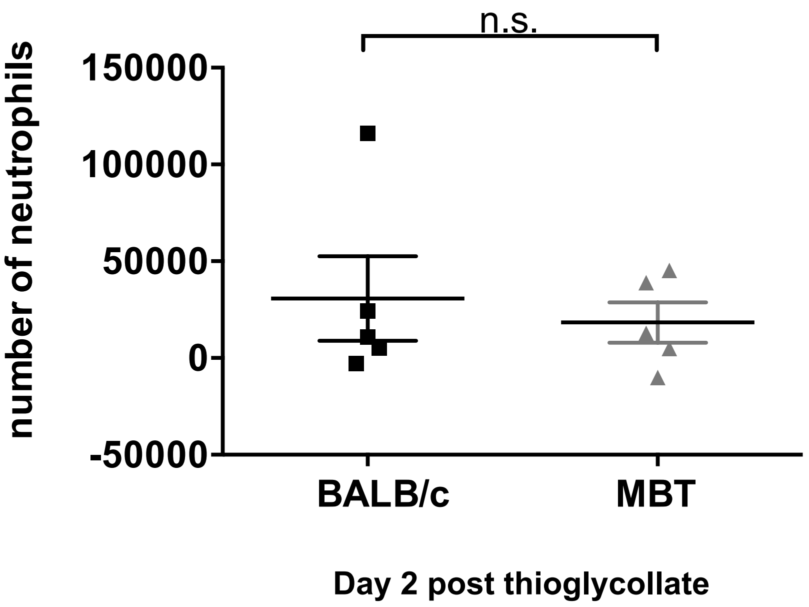

Supplementary Figure 6

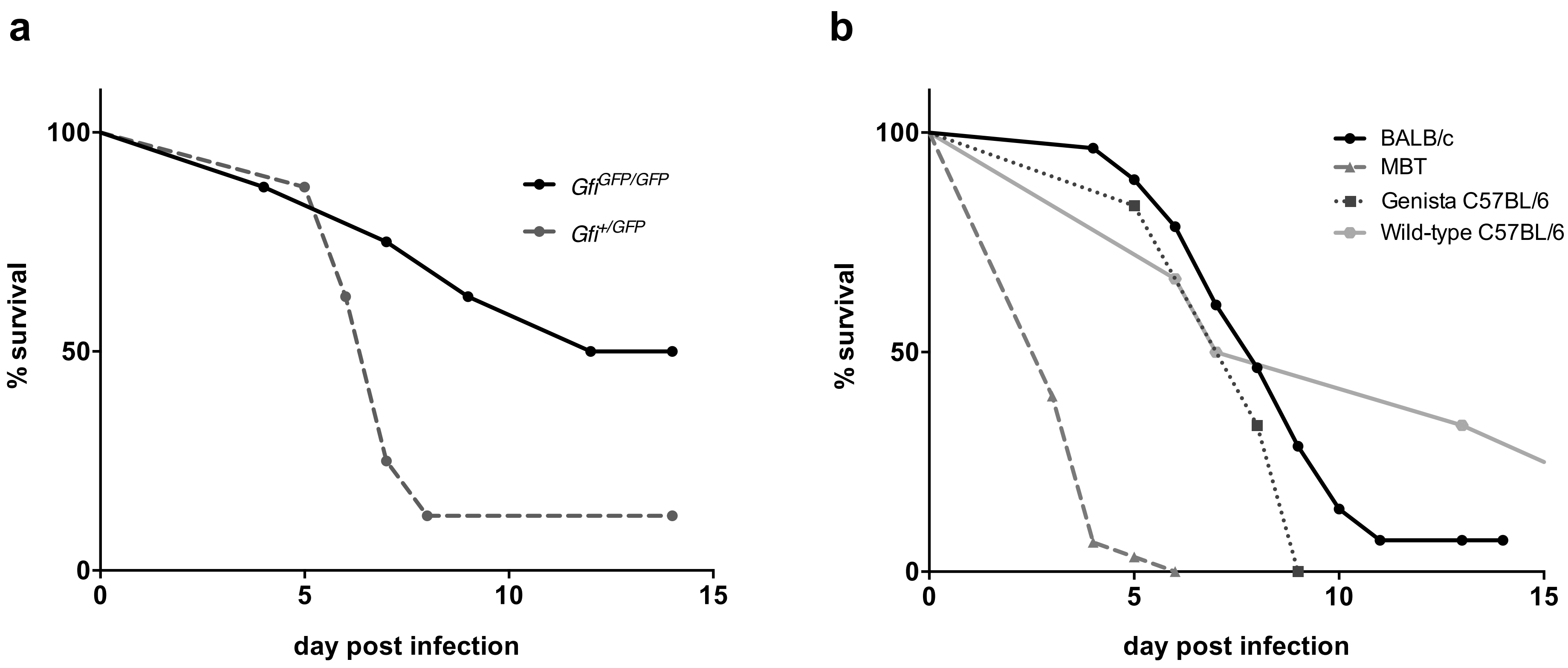

Constitutive neutropenia has not effect on survival of infected mutant and wild type C57BL/6 mice.

Supplementary Figure 7

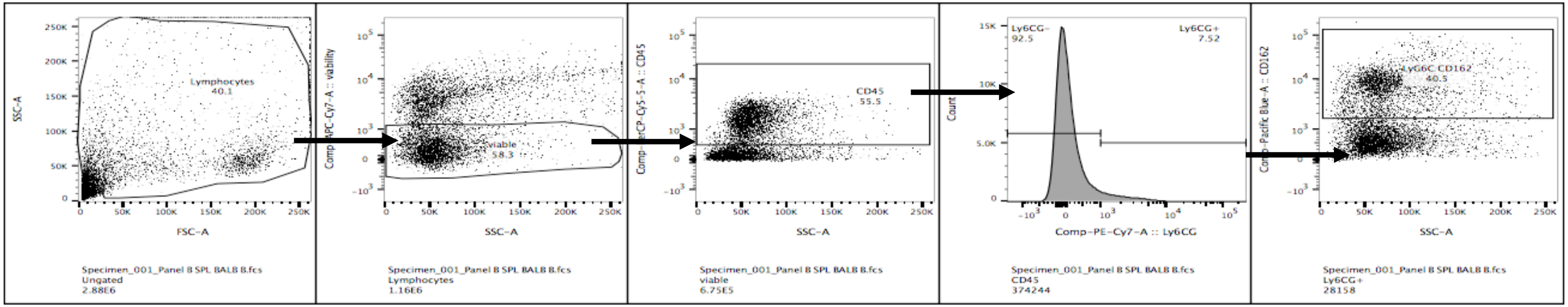

Typical gating scheme used to identify immune cell subpopulations.

S1 Table. Antibodies used for staining.

Antibody	Target Epitope/CellsCells	FluorochromeFluorochrome	Clone	Manufacturer
CD45	Leukocytes	PerCP-Cy5.5, PE-Cy7	30-F11	BD Biosciences
CD335	NK cells	PE, BV421	29A1.4	BD Biosciences
Ly6G/Ly6C	Neutrophils, Monocytes, Macrophages	PE-Cy7, FITC, BV421	RB6-8C5	BD Biosciences
CD11b	Macrophages, DCs	PE, PerCP-Cy5.5, APC	M1/70	BD Biosciences
CD11c	DCs, Macrophages	PE, PE-Cy7, APC, APC-Cy7	HL3	BD Biosciences
CD317	pDCs	PE, APC	eBio129c,eBio927	eBioscience
CD19	B-cells	APC	1D3	BD Biosciences
CD3e	T-cells	PE-Cy7, APC-Cy7, APC	145-2C11	eBioscience, BD Biosciences
CD8a	Killer T-cells	FITC, PerCP-Cy5.5, APC	53-6.7	BD Biosciences
CD4	Helper T-cells	PE-Cy7, APC, APC-Cy7	GK1.5	BD Biosciences, eBioscience
*Rabbit anti-N RVFV antigen	RVFV virus particles	purified	IH918	Institut Pasteur
Anti-rabbit IgG	-	FITC, PE	-	BD Biosciences
Fixable viability	Non-viable cells	eFluor 780	-	BD Biosciences
*Caspase-3	Apoptotic cells	PE	C92.605	BD Biosciences
IFNAR1	Interferon alpha/beta receptor	PE	MAR1-5A3	eBioscience
*IFN-gamma	Interferon –gamma	Alexa647	XMG1.2	BD Biosciences
CD162	PSGL-1	PE, AlexaFlour 647	2PH1	BD Biosciences

*intracellular stain
